# Expression of lignocellulolytic enzymes in *Pichia pastoris*

**DOI:** 10.1186/1475-2859-11-61

**Published:** 2012-05-14

**Authors:** Andrea Mellitzer, Roland Weis, Anton Glieder, Karlheinz Flicker

**Affiliations:** 1 Institute of Molecular Biotechnology, Graz University of Technology, Graz, Austria; 2 ACIB GmbH, Austrian Centre of Industrial Biotechnology, Graz, Austria; 3 VTU Technology GmbH, Grambach, Austria

**Keywords:** xylanase, mannanase, cellobiohydrolase, synthetic gene, synthetic promoter, quantitative real time PCR, *Pichia pastoris*, fermentation, strain development

## Abstract

**Background:**

Sustainable utilization of plant biomass as renewable source for fuels and chemical building blocks requires a complex mixture of diverse enzymes, including hydrolases which comprise the largest class of lignocellulolytic enzymes. These enzymes need to be available in large amounts at a low price to allow sustainable and economic biotechnological processes.

Over the past years *Pichia pastoris* has become an attractive host for the cost-efficient production and engineering of heterologous (eukaryotic) proteins due to several advantages.

**Results:**

In this paper codon optimized genes and synthetic alcohol oxidase 1 promoter variants were used to generate *Pichia pastoris* strains which individually expressed cellobiohydrolase 1, cellobiohydrolase 2 and beta-mannanase from *Trichoderma reesei* and xylanase A from *Thermomyces lanuginosus*. For three of these enzymes we could develop strains capable of secreting gram quantities of enzyme per liter in fed-batch cultivations. Additionally, we compared our achieved yields of secreted enzymes and the corresponding activities to literature data.

**Conclusion:**

In our experiments we could clearly show the importance of gene optimization and strain characterization for successfully improving secretion levels. We also present a basic guideline how to correctly interpret the interplay of promoter strength and gene dosage for a successful improvement of the secretory production of lignocellulolytic enzymes in *Pichia pastoris*.

## Background

Although *Pichia pastoris* is a relatively simple eukaryotic organism it can perform many posttranslational modifications such as glycosylation, disulfide bond formation, and proteolytic processing [1]. Therefore, *Pichia* serves as an interesting alternative to other (more difficult to handle) fungal secretory expression systems that are used to produce lignocellulolytic enzymes and other eukaryotic proteins which typically require post-translational modifications for correct folding, stability and activity. The recalcitrant and complex nature of lignocellulosics [2] affords the application of complex enzyme mixtures for efficient hydrolysis of these renewable sources. Consequently, for a sustainable production of fuels, chemical building blocks, and functional macromolecules from plant biomass a multitude of different enzymes is needed. To produce all these enzymes and variants thereof, production strains which can be handled and engineered in a simple way need to be generated. Therefore, being a well- described and widely applied expression host [3]*P. pastoris* was the first choice for the heterologous expression of the selected target proteins. Furthermore, in contrast to many other eukaryotic expression systems *P. pastoris* secretes no endogenous lignocellulolytic enzymes in significant amounts [4]. Therefore, recombinant *Pichia* strains can provide almost pure heterologous enzyme preparations without the need of extensive and costly downstream processing. In addition, simple media requirements and relative easy handling in bioreactors enable inexpensive large-scale cultivations of *Pichia*[5]. All these characteristic features of *Pichia* contribute to its high potential for cost reduction during the production of lignocellulolytic enzymes, particularly for application studies when only low- and medium-scale enzyme productions are required.

However, even though *Pichia pastoris* is a good host for the expression of heterologous proteins [3] there is still space for improvements on transcriptional [6,7] and (post-) translational level [8,9]. In this work we exemplify the impact of gene optimization on the overall expression level of lignocellulolytic enzymes in *Pichia pastoris*. Most genomes are heterogeneous in codon usage [10] and, accordingly, the codon bias of small subsets of genes may differ clearly from the average codon usage of the genome. To optimize protein coding sequences for enhanced protein expression in *Pichia pastoris* we use an in-house developed biased codon usage table [11]. This codon usage is biased towards the codons of selected, highly expressed [12,13] endogenous and heterologous genes when the AOX1 promoter and methanol were used for induction in *Pichia pastoris*. In addition to gene optimization, enzyme expression can be influenced on a transcriptional level by varying copy numbers of the integrated expression cassettes and by the choice of the promoter. So far the wildtype AOX1 promoter (P(AOX1)) and, to a certain extent, the GAP promoter (P(GAP)) were mostly used for heterologous protein production in *Pichia pastoris*[3]. However, since the P(GAP) is strong and constitutive it is not a good choice for production of physiologically problematic or cytotoxic proteins [14]. In contrast, the P(AOX1) is even stronger but also tightly regulated. Nonetheless, for some heterologous proteins the high transcript level generated by P(AOX1) can overload the cellular post-translational machinery, resulting in misfolded, unprocessed, or mislocalized proteins that can trigger a complex cellular response known as the unfolded protein response [15,16]. To overcome these disadvantages of the wild-type GAP and AOX1 promoter a library of promoters based on the wild-type P(AOX1) was previously generated [6]. The distinct properties of these novel promoters regulate the transcript level of target mRNA in response to the available carbon source level and type and concomitantly achieve a fine-tuned protein expression in *Pichia pastoris*.

The aim of the present study was to show the functional expression of lignocellulolytic enzymes in *Pichia pastoris* at high quantities and investigating the effect of gene optimization and of alternate promoters on the expression level of these enzymes. Our expression studies highlight basic principles for designing suitable expression constructs and for successful strain development for different cellulolytic enzymes. For this study *Trichoderma reesei* cellobiohydrolase 1 and 2 (*Tr*CBH1 and *Tr*CBH2) and beta-mannanase (*Tr*bMan), and *Thermomyces lanuginosus* xylanase A (*Tl*XynA) were chosen as target enzymes.

## Results and discussion

The goal of this study was to evaluate the potential of *Pichia pastoris* to express lignocellulolytic enzymes. In particular, we improved the expression of selected (hemi-) cellulases by codon optimization of the target genes, investigated the effect of promoter choice, and characterized the performance of selected producer strains in small-scale bioreactors. This characterization also included the effects of multi-copy integration on the productivity for the selected target enzymes.

To investigate the effect of different methods for codon optimization three different gene variants of *Trichoderma reesei* cellobiohydrolase 2 (*Tr*CBH2) were employed; the native gene variant (*Tr*CBH2-wt), a gene variant with optimized codon pairs by a commercial supplier (*Tr*CBH2-CP) and an in-house-optimized variant (*Tr*CBH2-HM). For the in-house design a codon usage table [17] derived from genes which are highly expressed in *Pichia pastoris* in methanol containing media was used.

**Figure 1:**
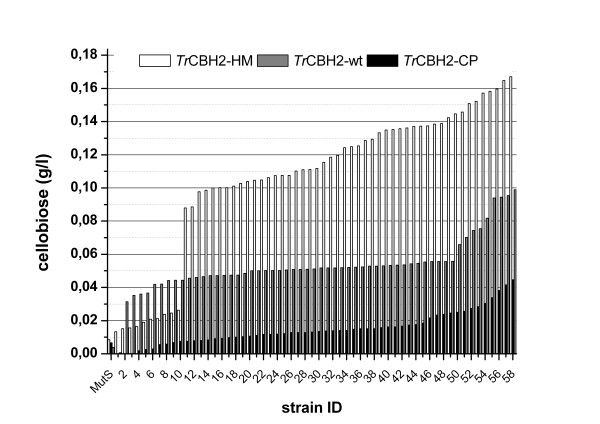
**Activity landscapes of individual*P. pastoris*transformants expressing three different*Tr*CBH2 variants controlled by P(AOX1).** Codon pair optimized sequence in black (CP), wt sequence in grey, high CAI codons for methanol induced gene expression in white (HM) [11]. Released cellobiose concentration is represented in bars. The untransformed strain *P. pastoris* CBS7435 Mut^S^ was used as negative control

The effects of gene optimization and promoter type were characterized by comparing activity landscapes of different strains (Figure 1). For this purpose *P. pastoris* strains were cultivated in 96 deep-well plates according to [18] and subsequently screened for lignocellulolytic activities using a reducing sugar assay that was recently adapted to high-throughput [19]. Owing to the low standard deviation of this assay, the detected changes in the activity landscapes mainly reflect actual changes in the expression level [19]. These differences can either be due to the number of integrated expression cassettes or caused by specific effects of the individual gene variants. Figure 1 shows enzyme activity landscapes of *Tr*CBH2-wt and the two differently optimized *Tr*CBH2 gene variants which have all been separately incorporated into the same expression vector and host. Stable integration of expression cassettes into the *Pichia pastoris* genome is generally based on homologous recombination but can also be an effect of non-homologous end-joining (NHEJ). Depending on the length, type and structure of the homologous flanking regions, untargeted (random) genome integration mediated by NHEJ becomes prevalent over locus-specific targeting (own observation for our vector system). Therefore, expression levels may be influenced not only by the number of integrated gene copies [20] but also by the integration locus which influences the transcript levels of the integrated genes. Our results demonstrate a clear effect of gene optimization on expression level. This is corroborated by the fact that our interpretation of expression level does not rely on a single observation but is averaged over a whole activity landscape of many individual transformants (Figure 1). This could be substantiated by reliably proving low copy numbers among differently optimized genes, in order to get a decent comparability of the influence. The 2-fold increase in expression level of *Tr*CBH2-HM compared to *Tr*CBH2-wt suggests a more efficient transcription and/or translation of this variant in *P. pastoris*. Contrary to this, the gene optimized by the commercial service using codon pair optimization, *Tr*CBH2-CP, showed a 2-fold lower expression level than *Tr*CBH2-wt*. B*eing originally designed to assist co-translational protein folding [21] of multi-domain proteins we expected the optimization based on codon pair signaling to show improved expression for the two-domain enzyme *Tr*CBH2. However, as we observed the opposite effect for *Tr*CBH2-CP we speculate that the bottleneck of *Tr*CBH2 expression is rather on transcriptional level than on the posttranslational level of protein folding. Summarizing, the optimized gene variant *Tr*CBH2-HM was superior to all other variants under the tested methanol-inducing conditions. This suggests that preferring codons with a high codon adaptation index (CAI) for highly expressed proteins under methanol inducing conditions is a good choice for *Tr*CBH2.

Especially for secreted proteins the level of expression strongly depends on the number of integrated expression cassettes. Therefore, often the production efficiency of a strain can be predicted by quantifying the number of genome-integrated expression cassettes (copy number, CN) [15,20,22,23]. In *P. pastoris* an initial (linear) positive correlation between copy number and productivity that stagnates at a defined upper limit can be observed [20,22]. Furthermore, in some cases also a loss of productivity above a certain number of integrated copies has been described [15,23]. In fact, high mRNA-levels caused by strong promoters or by high numbers of the expression cassettes can overload the folding and secretion machinery of the host. Depending on the protein this can entail an accumulation of unfolded proteins which triggers dedicated signaling pathways, commonly known as the unfolded protein response [24]. For comparative studies it is, therefore, essential to characterize the strains with regard to their copy numbers. Doing so will also allow the separation of promoter and/or copy number related effects on expression levels.

**Figure 2:**
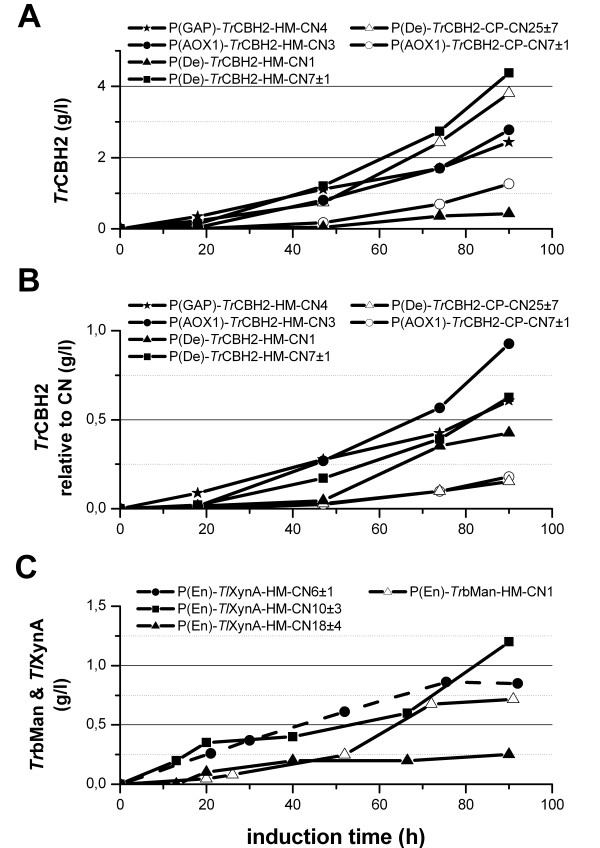
**Time-course of protein concentration during fed-batch cultivations.** Gene sequences were optimized either by codon pair optimization (CP) [21] or by applying the high CAI codons for methanol induced genes (HM) [11]. Copy numbers (CN) are specified in the legend. Panel **A**: Expression of differently codon optimized *Tr*CBH2 variants under the control of P(AOX1), P(GAP) or the synthetic promoter P(De) [6]. HM optimized variants (closed symbol), CP optimized variants (open symbol). Panel **B**: Time-course of *Tr*CBH2 expression normalized to copy number. Legend labeling see panel A. Panel **C**: Expression of *Tr*bMan (open symbol) and *Tl*XynA (closed symbol) under the control of P(En) [6]. For virtual gels of the protein yields during the fermentation runs please refer to Additional file 1 and the raw data can be found in Additional file 2

**Figure 3:**
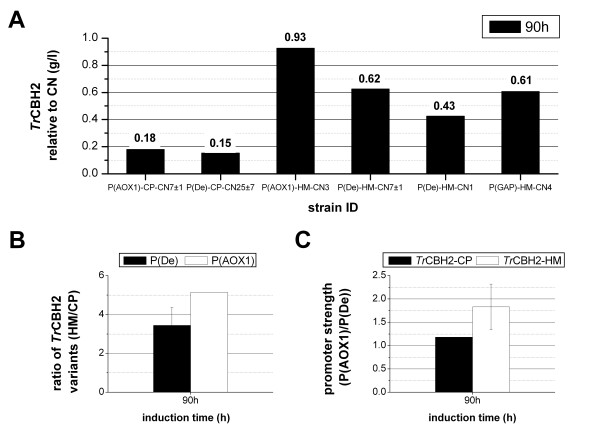
**Comparison of*Tr*CBH2 gene/ promoter variants normalized to gene copy numbers (CN).** Panel **A**: Protein concentration of T*r*CBH2 fed-batch cultivations normalized by CN after 90 h of induction. Panel **B**: Relative ratios of the expression levels of the different gene optimization variants (HM/CP) under the control of P(AOX1) (white bar) and P(De) (black bar). Panel **C**: Relative ratios of the normalized expression levels of the different methanol inducible promoters (P(AOX1)/P(De)) expressing either *Tr*CBH2-CP (black bars) or *Tr*CBH2-HM (white bar) after 90 h of induction. Please refer to Additional file 2 for the raw data and Results and discussion section for more detailed information

To determine the individual expression levels of *Tr*CBH2 expressing *P. pastoris* strains under bioreactor conditions we selected suitable strains based on initial micro-scale screenings in 96 deep-well plates and by quantitative gene copy number determination using qRT-PCR. Gene expression was driven either by the wild- type promoters P(AOX1) and P(GAP) or by synthetic promoter variants. These synthetic promoter variants are part of a newly generated promoter library based on P(AOX1) which was designed to fine-tune protein expression in *Pichia pastoris*[6]. With regard to their particular regulatory features two of these synthetic promoters were chosen to be tested in this study, namely P(En) and P(De). In the original publication by Hartner *et al.*[6] P(En) showed similar low expression of the reporter protein green fluorescent protein (GFP) under derepressed conditions but increased expression up to 166%, when compared to the wild-type promoter P(AOX1) on single copy level after 0 h and 72 h of methanol induction, respectively. P(De) showed more than 4-fold higher GFP fluorescence intensity under derepressed conditions but decreased expression down to 55%, if compared to the wild-type promoter P(AOX1) on single copy level after 0 h and 72 h of methanol induction, respectively. Even though, GFP expression driven by P(De) resulted in decreased protein production it was shown that this promoter was favorable for difficult to secrete proteins such as horseradish peroxidase (HRP). The overall productivity in fed-batch cultivations of HRP expression driven by P(De) was significantly higher than compared to the overall productivity of HRP expression driven by P(AOX1) [6]. In Figure 2A the time-courses of protein concentrations in the supernatants during fed-batch cultivations of *Tr*CBH2 are compared. Figure 2A shows that the strain P(De)-*Tr*CBH2-CP-CN25 ± 7 which harbors about 25 expression cassettes achieved around 4 g/l of *Tr*CBH2. This is comparable to the expression of P(De)-*Tr*CBH2-HM-CN7 ± 1 which is optimized using our in-house HM method. It can also be seen from Figure 2B that our in-house gene optimization method HM outperforms that of the commercial supplier (compare also Figures 1 and 3B). Although different promoters were used the expression of P(De)-*Tr*CBH2-CP-CN25 ± 7 and P(AOX1)-*Tr*CBH2-CP-CN7 ± 1 normalized to the same level suggesting that a linear correlation of expression independent of promoter type up to a CN of 25 exists for the CP optimization. Based on these data and on data from literature [20,22] we observed two properties for *Tr*CBH2 expression. Firstly, using the AOX1 promoter variant P(De) we observed a positive initial (linear) correlation up to at least 7 copies between copy number and productivity. Secondly, gene optimization with our in-house method results in higher expression level at low copy numbers.

As observed in the micro-scale screening (Figure 1) *Tr*CBH2-HM led to a higher expression level than *Tr*CBH2-CP (Figure 3) also in fed-batch cultivations. This effect was even more pronounced for the expression regulated by P(AOX1) with a 5-fold improvement of *Tr*CBH2-HM over *Tr*CBH2-CP (Figure 3B). In contrast, for the P(De)-*Tr*CBH2 variants we only observed a 3-fold improvement (Figure 3B) which can be explained by the lower promoter strength of P(De) as described previously [6]. In addition, the relative ratios of the different gene optimization variants (Figure 3B) and the relative ratios of the different gene promoter variants (Figure 3C) allow also a better comparison between the methanol inducible promoters P(AOX1) and P(De). (Figure 3C) shows that the strong methanol-inducible P(AOX1) increases expression of *Tr*CBH2-HM around 1.7-fold compared to expression under the control of P(De). For *Tr*CBH2-CP expression under the control of different promoters only a 1.2-fold improvement can be seen (Figure 3C). These results clearly indicate for *Tr*CBH2 expression that the codon optimization which is based on the codon bias of highly transcribed genes under methanol-inducing conditions gives even higher expression when a strong methanol-inducible promoter is employed.

**Figure 4:**
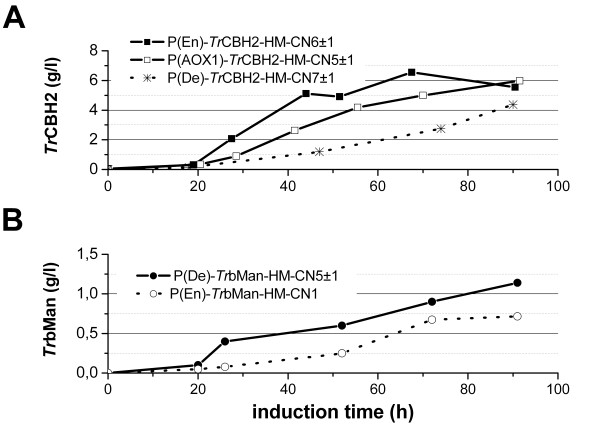
**Time-course of protein concentration during fed-batch cultivations.** Gene sequences were optimized by applying the high CAI codons for methanol induced genes (HM) [11]. Copy numbers (CN) are specified in the legend. Dotted lines indicate previously obtained results (compare Figure 2). Panel **A**: *Tr*CBH2 expression under the control of P(AOX1) or the synthetic promoters P(En) or P(De) [6]. Panel **B**: *Tr*bMan expression under the control of P(De) and P(En). For a visual representation of the protein yields during the fermentation runs please refer to the virtual gels presented in Additional file 1 and the raw data can be found in Additional file 2

After 90 h of induction the single copy expression level of P(De)-*Tr*CBH2-HM-CN1 is approximately 0.43 g/l (Figure 2B) whereas a higher single copy expression level of about 0.930 g/l (normalized to CN) can be calculated for P(AOX1)-*Tr*CBH2-HM-CN3. Based on these data, P(AOX1) gives an around 2-fold higher expression level than P(De). Although the strain P(De)-*Tr*CBH2-HM-CN7 ± 1 performed best under the tested MeOH-inducing conditions our results, based on the normalized data, indicate that strong methanol-inducible promoters such as P(AOX1) or the even stronger methanol-inducible P(En) [6] can further increase the expression of *Tr*CBH2. To verify this hypothesis on fermenter scale we decided to screen for higher copy number strains expressing *Tr*CBH2 under the control of P(AOX1) and P(En). As seen in Figure 4/A the selected strains with increased copy numbers P(AOX1)-*Tr*CBH2-HM-CN5 ± 1 and P(En)-*Tr*CBH2-HM-CN6 ± 1 indeed produced significantly more protein over the whole induction period than the best strain of the first fermentation P(De)-*Tr*CBH2-HM-CN7 ± 1. Within the first 70 h of induction the productivity of P(En)-*Tr*CBH2-HM-CN6 ± 1 was higher than the productivity of P(AOX1)-*Tr*CBH2-HM-CN5 ± 1. This confirms the results of the previously reported GFP expression experiments [6] using an improved synthetic AOX1 promoter variant. The final protein yield of both strains was comparable at around 6 g/l. Summarizing, using strong methanol inducible promoters in combination with high copy numbers of genes that are optimized to a high CAI for highly expressed proteins under methanol induction can further increase the yield of *Tr*CBH2. Moreover, we showed that pre-selection of strains using micro-scale screenings and further strain characterization using qRT-PCR for copy number determination is a useful tool to reduce bioreactor cultivations to a reasonable number.

Although *Trichoderma reesei* typically can produce more than 100 g/l of cellulases [25], individual enzymes such as *Tr*CBH2 are expressed in much lower quantities (10-15%) [26]. Table 1 gives an overview of published expression yields and activities of the different lignocellulolytic enzymes in different host systems. So far, Miettinen-Oinonen *et al*. achieved the highest protein concentration of 0.7 g/l *Tr*CBH2 in *T. reesei* strains cultivated in shake flasks [27] which is around 9-fold less than compared to our highest cellobiohydrolase concentration in *Pichia pastoris* bioreactor cultures. For other heterologous host systems such as *S. cerevisiae*[28] and *S. pombe*[29] even lower *Tr*CBH2 concentrations in the range of 0.1 g/l have been reported. Regarding the specific activities of *Tr*CBH2, we obtained 3.04 U/mg on Avicel, 5.30 U/mg on PASC and 1.51 U/mg on CMC whereas 2.52 U/mg on PASC and 0.09 U/mg on CMC have been reported for the *S. pombe* system [29].

**Table 1:** Protein yields and enzymatic activities of expressed lignocellulolytic enzymes

**Enzyme**	**Host**	**Yield **	**Activity**	**Spec. Activity**	**Reference**
		**(g/l)**	**(U/ml)**	**(U/mg)**	
*Tr*CBH2	*P. pastoris*^B^	5.984	18.21^1^	3.04	this study
			31.70^2^	5.30	
			9.05^3^	1.51	
*Tr*CBH2	*S. cerevisiae*^B^	0.1	^n.d.^	^n.d.^	[28]
*Tr*CBH2	*T. reesei*^A^	0.7	^n.d.^	^n.d.^	[27]
*Tr*CBH2	*S. pombe*^A^	0.115	0.29^2^	2.52	[29]
			0.01^3^	0.09	
*Tr*bMan	*P. pastoris*^B^	1.142	109^4^	95.45	this study
*Tr*bMan	*S. cerevisiae*^A^	0.000150	0.01^4^	66.67	[30]
*Tr*bMan	*T. reesei*^A^	^n.d.^	85.85^4^	^n.d.^	[30]
*Tr*bMan	*T. reesei*^A^	^n.d.^	1.8^4^	^n.d.^	[31]
*Tl*XynA	*P. pastoris*^B^	1.2	138^5^	115.00	this study
*Tl*XynA	*P. pastoris*^A^	0.148	40.2^5^	271.62	[32]
*Tl*XynA	*T. lanuginosus*^A^	0.270	88.5^5^	327.78	[32]
*Tl*XynA	*P. pastoris*^A^	0.236	26.8^5^	113.56	[33]

^A^ shake flask cultivation.

^B^ fed-batch cultivation.

^n.d^ not determined.

^1^ Avicel.

^2^ phosphoric acid-swollen cellulose (PASC).

^3^ carboxymethylcellulose (CMC).

^4^ locust bean gum.

^5^ birchwood xylan.

To evaluate *P. pastoris’s* capability for expressing various other lignocellulolytic enzymes we also expressed xylanase A from *Thermomyces lanuginosus* (*Tl*XynA), beta-mannase from *Trichoderma reesei* (*Tr*bMan) and cellobiohydrolase 1 from *Trichoderma reesei* (*Tr*CBH1). All genes were optimized using the in-house codon usage table and subsequently cloned downstream of the synthetic promoter P(En) [6]. *Tr*bMan was also cloned downstream of the constitutive P(GAP) promoter. For *Tl*XynA only the strong promoter P(En) was selected to push its already proven high expression in *Pichia pastoris* with the native AOX1 promoter [34]. Similar to the experiments for *Tr*CBH2 high-throughput deep-well plate screenings were performed with the adapted pHBAH-assay and the CNs were determined by qRT-PCR. Five *Tr*bMan P(En) strains harboring 1, 4, 6 ± 1, 16 ± 4 and 39 ± 9 copies, one single copy strain under the control of P(GAP) and three *Tl*XynA P(En) strains with 6 ± 1, 10 ± 3, and 18 ± 4 copies were fed-batch cultivated. Although we successfully expressed *Tr*CBH1 in the micro-scale screening bioreactor fermentations yielded similar low protein concentrations for heterologously protein expression in the range of a few mg per liter as previously reported in literature [28,35,36]. Therefore, those strains were not characterized in more detail for this paper.

Although the initial micro-scale screening revealed expression of *Tr*bMan under the control of P(AOX1) and P(GAP) over a broad range of CNs only the single copy strain of *Tr*bMan P(En) successfully produced *Tr*bMan in the bioreactor (Figure 2). All other P(En) regulated strains with more than one copy had major growth problems shortly after induction resulting in attenuated growth (data not shown) when grown under our standard cultivation conditions. In contrast, the P(En)-*Tr*bMan-HM-CN1 strain showed normal growth after recovering from an initial cessation of growth post methanol-induction (data not shown). Under constitutive expression of *Tr*bMan using P(GAP) the growth rate was slowed down and no *Tr*bMan was produced even though just a single expression cassette was integrated into the *Pichia* genome (data not shown). This could be a further example of the potentially cyto-toxic effects [14] of constitutive heterologous protein expression with P(GAP) in *P. pastoris*. In addition, *Tr*bMan seems to be generally difficult to express in yeasts under constitutive promoters. As an example, *Tr*bMan was only produced at a level of 0.150 mg/l in *Saccharomyces cerevisiae*[30] under the control of the constitutive phosphoglycerate kinase (PGK) promoter. Our experiments revealed that the molecular weight ratio of glycosylated and deglycosylated *Tr*bMan was about 4 (determined by capillary electrophoresis, data not shown). Therefore, hyper-glycosylation of *Tr*bMan in *P. pastoris* might be another problem for expression.

To test if a weaker methanol-inducible promoter can increase the productivity we also tested the synthetic promoter P(De) for *Tr*bMan. As seen in Figure 4B, over the whole induction period the strain P(De)-*Tr*bMan-HM-CN5 ± 1 achieved significantly more protein than compared to P(En)-*Tr*bMan-HM-CN1 (Figure 4B). As previously seen in deep well experiments [6] P(De) has a weak onset of expression during the glucose depletion-derepression phase which could also be presumed for bioreactor cultivations. Based on that, we further assume that a weaker onset leads to a better adaptation of the *Pichia* system for the production of *Tr*bMan. In addition, it was recently shown that expression under the control of P(De) can result in positive effects on cell physiology compared to expression under the control of P(AOX1) [37]. Consequently, P(De)-*Tr*bMan-HM-CN5 ± 1 was capable of producing 1.142 g/l of protein (Figure 4B) devoid of any directly observable growth problems during fermentation (data not shown). The obtained yield of 1.142 g/l of *Tr*bMan is ~7600-fold higher than the so far highest reported heterologous yield of 0.150 mg/l expressed in *Saccharomyces cerevisiae*[30] (Table 1). The activity of *Tr*bMan expressed in our study was 109 U/ml using locust bean gum as substrate. This was similar to the results of Stalbrand *et al*. [30] who obtained ~86 U/ml in *Trichoderma reesei* shake flask cultures. However, *T. reesei* shake flask cultivations performed by Hagglund *et al*. [31] showed an activity of 1.8 U/ml. This value is about 60 times less than compared to our results. Comparing heterologously expressed *Tr*bMan our obtained activity of 109 U/ml is approximately 11000-fold higher than the 0.01 U/ml expressed in *S. cerevisiae*[30]. Regarding the specific activities for *Tr*bMan, we achieved 95.4 U/mg compared to 66.7 U/mg for *Tr*bMan expressed in *S. cerevisiae*[30] (see Table 1) which usually shows an even higher tendency for hyper mannosylations that could limit the activity of *Tr*bMan*.*

For the fourth target, *Tl*XynA, fed-batch bioreactor cultivation of *Pichia* strains regulated by P(En) and harboring 6 or 10 integrated expression cassettes produced around 1 g/l whereas one strain with 18 integrated expression cassettes showed a reduced protein concentration of 0.25 g/l (Figure 2). As mentioned before, such negative correlation at higher copy numbers and productivity had already been described in literature for other proteins expressed in *Pichia*[15,23].

Our yield of 1.2 g/l *Tl*XynA represents a 5 to 8-fold increase in yield compared to earlier expression studies in *Pichia pastoris* by Damaso *et. al.*[32] and Gaffney *et. al.*[33], respectively. Compared to *T. lanuginosus* shake flask cultures we achieved about 4 times more protein than reported before in [32] (Table 1). Our obtained specific activity of 115.00 U/mg was similar to the specific activities of 113.56 U/mg and 271.62 U/mg that were obtained by *Pichia pastoris*, [33] and [32] respectively. The specific activity of homologously expressed *Tl*XynA of 327.8 U/mg [32] was approximately 3-fold higher than compared to our obtained values. The comparison to homologously expressed *Tl*XynA indicates that the enzyme produced in *P. pastoris* showed lower specific activity although total volumetric yields were higher.

Generally, we speculate that the variation in specific activities of all enzymes could predominantly be attributed to the different glycosylation pattern that is produced by *P. pastoris*[38]. This phenomenon has already been described in literature *e.g.* by Macauly-Patrick *et.al.*[14].

Unfortunately, there are only limited bioreactor cultivations reported for *Tr*CBH2, *Tr*bMan, and *Tl*XynA, therefore, above made direct comparison of bioreactor results to published shake flask expression experiments are biased. However, we can still conclude that homologous expression yielded the highest specific activities but not necessarily the highest total protein yields. Although *P. pastoris* is an excellent host for achieving high protein concentrations heterologous expression can also influence the activity of the expressed enzymes. Nevertheless, comparing the calculated specific activities from Table 1 there is a general trend that the specific activities of the enzymes produced by *P. pastoris* are in the range or even higher than the specific activities of the same enzymes expressed in other heterologous hosts. This makes *Pichia* a good compromise for the expression of high quantities of enzymes with relatively high specific activities. Furthermore, it also shows the possible relevance of host strain glyco-engineering for industrial enzyme production as it already has for the production of biologically active pharmaceutical proteins.

## Conclusions

We have successfully constructed *P. pastoris* strains capable of producing maximum protein concentrations of 1.142 g/l *Tr*bMan, 6.55 g/l *Tr*CBH2, and 1.2 g/l *Tl*XynA in fed-batch bioreactor cultivations. Moreover, we showed that suitable codon optimization of the target genes helps to increase heterologous protein production by *P. pastoris*, thus providing a simple way of increasing heterologous protein production for individual enzymes.

Furthermore, we emphasize the importance of transcript level optimization by alternative promoters and gene dosage (numbers of integrated gene copies) for expression optimization. This was particularly evident for the functional expression of *Tr*bMan. The strong constitutive and methanol inducible promoters P(GAP) and, P(AOX1) respectively, secreted no or less protein than the weaker synthetic promoter P(De).

Basically there are three classes of genes (A,B,C) with varying dependence of yields of active proteins in relation to copy numbers: For class A genes an increase in copy number to more than 10 copies has a positive effect on protein expression, as seen in the case of *Tr*CBH2. For class B genes the yield of active protein increases within the number of integrated copies up to a copy number of 2–10 and decreases with higher copy numbers, as seen in the case of *Tl*XynA. Finally, class C genes where yields of active protein get worse with increasing copy numbers, as seen in the case of *Tr*bMan. However, these effects definitely depend on the strength of the employed promoter as well as the gene encoding the respective target protein.

Our conclusions are based on a better understanding of promoter and/or copy number-related effects. Codon-optimized genes together with optimized promoters and numbers of integrated expression cassettes allowed us to develop *P. pastoris* strains producing high levels of lignocellulolytic enzymes. In combination with the high specific activities compared to the same enzymes expressed in other hosts, *Pichia* seems to be a good choice for the heterologous expression of individual lignocellulolytic enzymes.

## Methods

### Chemicals and Materials

Oligonucleotide primers were obtained from Integrated DNA Technologies (Leuven, Belgium). For plasmid isolation the GeneJET™ Plasmid Miniprep Kit of Fermentas (Burlington, Ontario, Canada) was used. All DNA-modifying enzymes were obtained from Fermentas GmbH (Burlington, Ontario, Canada). Chemicals were purchased if not stated otherwise from Becton, Dickinson and Company (Franklin Lakes, NJ, USA), Fresenius Kabi Austria (Graz, Austria), Carl Roth (Karlsruhe, Germany), and Sigma- Aldrich (St Louis, MO, USA). p-hydroxybenzoic acid hydrazide (order no. 54600) were obtained from Fluka (Hamburg, Deutschland). D-(+)-mannose and D-(+)-cellobiose were from Fluka, D-(+)-xylose from Sigma, D-(+)-glucose monohydrate from Carl Roth (Karlsruhe, Germany).

### Media

For *E. coli* standard LB-medium containing 25 μg/ml zeocin was used. YPD for *P. pastoris* contained 10 g/l yeast extract, 20 g/l peptone and 20 g/l glucose. For antibiotic selection 100 μg/ml zeocin were used. 15 g/l agar was added for plate media. Buffered minimal media BMD (1%), BMM2 and BMM10 consisted per liter of 200 ml 1 M potassium phosphate buffer (pH 6), 13.4 g yeast nitrogen base without amino acids, 0.0004 g/l biotin and 11 g/l glucose or 1 or 5% (v/v) methanol, respectively. All pre-cultures were prepared using YPhyD medium containing 20 g/l Phytone-Peptone, 10 g/l Bacto-Yeast Extract and 20 g/l glucose. BSM medium contained per liter CaSO_4__2H_2_O 0.47 g, K_2_SO_4_ 9.1 g, KOH 2.07 g, MgSO_4__7H_2_O 7.5 g, EDTA 0.6 g, H_3_PO_4_ (85%) 13.4 ml, Glycerol 40.0 g, NaCl 0.22 g and 4.35 ml PTM1. PTM1 Trace elements solution contained per liter 0.2 g Biotin, 6.0 g CuS0_4__5H_2_O, 0.09 g KI, 3.0 g MnSO_4__H_2_O, 0.2 g Na_2_MoO_4__2H_2_O, 0.02 g H_3_BO_3_, 0.5 g CoCl_2_, 42,2 g ZnSO_4__7H2O, 65 g Fe(II)SO_4__7H_2_O and 5 ml H_2_SO_4_. The fed-batch feed media were either 60% (w/w) Glycerol or concentrated MeOH and were supplemented with 12 ml/l PTM1 mineral salts solution.

### Construction of *P. pastoris* strains

The coding sequences of xylanase A from *Thermomyces lanuginosus* (*Tl*XynA) [UniProtKB/Swiss-Prot: O43097], beta-mannanase (*Tr*bMan) [UniProtKB/TrEMBL: Q99036], cellobiohydrolase 1 (*Tr*CBH1) [UniProtKB/Swiss-Prot: P62694] and cellobiohydrolase 2 (*Tr*CBH2) [UniProtKB/Swiss-Prot: P07987] from *Trichoderma reesei* inclusive of their natural secretion leaders were codon optimized for *P. pastoris* expression applying the Gene Designer software (DNA2.0, Menlo Park, CA, USA) based on an in-house developed codon bias [11]. The GC content was set to be between 40 and 60% without local peaks and restriction sites for cloning were avoided. In addition, one other variant of *Tr*CBH2 was ordered from a commercial supplier (CODA Genomics, Laguna Hills, CA) which optimized the genes based on the method of codon pair signaling [21]. The native DNA sequence was kindly provided by Frances H. Arnold. To further optimize translation all genes were cloned after a defined Kozak consensus sequence (gaaacg) [39]. The synthetic genes were cloned into the multiple cloning site of the *E. coli*/*P. pastoris* shuttle vector pPpB1 [11] via *EcoR*I/*Not*I restriction sites. The *Tr*CBH2 variants were cloned downstream of the wild type promoters P(GAP) and P(AOX1) and synthetic promoter variants with distinctly different regulation patterns were also included, namely P(En) and P(De). P(En) can be induced by methanol and showed increased GFP expression up to 166%, if compared to the wild-type promoter P(AOX1). P(De) can either be induced by methanol or under derepressed conditions as described by Hartner *et al*. [6]. *Tr*bMan and *Tl*XynA were cloned downstream of the synthetic promoter P(En) [6] and in addition *Tr*bMan was cloned downstream of P(GAP) and P(De). Plasmids were linearized with *Bgl*II, subsequently purified and concentrated using the Wizard_ SV Gel and PCR Cleanup System (Promega Corp.). Electro-competent *P. pastoris* CBS 7435 mut^S^ cells were prepared and transformed with 1- to 2 μg of the *Bgl*II-linearized pPpB1 vector construct according to Lin-Cereghino [40]. Transformants were plated on YPD-Zeocin (100 μg/ml Zeocin) agar plates and grown at 28°C for 48 h.

### Micro-scale cultivation and high-throughput screening

*P. pastoris* strains expressing *Tl*XynA*, Tr*bMan, and *Tr*CBH2 were cultivated in 96-deep well plates as described by Weis *et al*[18]. Incubation was done in shakers (INFORS Multitron, Bottmingen, Switzerland) at 28°C, 320 rpm, and 80% relative humidity. After an initial batch phase for 60 h on 1% glucose the cultures were induced with 0.5% of methanol for a total of 72 h (additional supplementations to 0.5% methanol were added after 12, 36 and 60 h of the first induction with methanol). After induction the cells were pelleted at 4000 rpm and enzymatic activities were determined in the supernatants using the pHBAH-assay as previously described by Mellitzer *et al*. [19]. Substrate conversions were performed in 50 mM citrate buffer containing appropriate substrate for each enzyme (either suspensions of 1% Avicel®, 0.25% PASC or solutions of 0.25% CMC, 0.5% xylan or 0.2% locust bean gum) at 50°C (*Tr*CBH2, *Tr*CBH1 and *Tr*bMan) or at 59°C (*Tl*XynA). The incubation time was 2 h for the cellobiohydrolases and 20 min for *Tl*XynA and *Tr*bMan. For the subsequent reducing sugar assay 50 μL of the substrate reaction (or, in the case of the standard sugars, appropriate dilutions of the reducing sugars) were pipetted into 150 μl of the pHBAH working solution in a 96-well PCR plate. The plate was sealed and incubated at 95°C for 5 min and then cooled to 4°C. 150 μl of the assay samples were transferred to a new micro-titer-plate and the absorption measured at 410 nm in a SPECTRA MAX Plus384 plate reader (Molecular Devices Corp., Sunnyvale, CA, USA). For exact quantification of reducing sugars a standard curve of the respective reducing sugar (0–1 mg/ml) was included on each plate. Activity units for the expressed enzymes refer to the amount of released reducing sugar over time and correspond to the standard IUPAC definition μM/min.

### Copy number determination by quantitative real-time PCR

Copy numbers of integrated expression cassettes in the *Pichia* genome were determined using quantitative real-time PCR (qRT-PCR) as described by Abad *et al.*[17].

### Fed-batch cultivations of *Pichia pastoris* strains

Pre-cultures of individual strains were grown in 50 and 200 ml YPhyD in wide-necked, baffled shake flasks at 120 rpm at 28°C. Each fermenter (Infors Multifors system (Infors AG, Bottmingen, Switzerland)) containing 450 ml BSM-media (pH 5.0) was inoculated from the pre-culture to an OD600 of 2.0. During the batch phase *P. pastoris* was grown on glycerol (4%) at 28°C. At the beginning of the glycerol feeding phase the temperature was decreased to 24°C. For methanol-fed cultures, the fed-batch phase was started upon depletion of initial glycerol with 16 g/(l*h) glycerol feed solution followed by methanol induction. In the early stages, the methanol-feed was set to 2 g/(l*h) and was gradually increased within the next 70 h to 6 g/(l*h). Likewise, the glycerol-feed was phased down during the first hour of methanol induction to 0 g/(l*h). Dissolved oxygen was set to 30% throughout the whole process. After 91.5 h of methanol induction the fermentations were stopped. For glycerol-fed strains, the batch phase was directly followed by a constant glycerol-feed with 6 g/(l*h). Protein concentrations were determined by micro-fluidic capillary electrophoresis (CE) using fluorescence detection (Caliper GXII System, Hopkinton, USA). Standard deviations of this robust system are usually below 10%, even at high protein loads (exemplified in Additional file 3). Therefore, just single measurements of every sample were performed. More specifically, proteins were quantified by calibrating the integrated areas of the protein-specific peaks in the electropherograms to an external reference protein standard (BSA) of known concentration. For glycosylated proteins, peak areas of diluted deglycosylated samples were compared to those of untreated samples to compensate for glycosylation-related differences in quantification (a comparison of glycosylated and non-glycosylated enzyme samples is exemplified for *Tr*bMan in Additional file 4). The dilutions of samples were in a range to give peak areas of the samples that were comparable to those of the reference protein standard. Importantly, the absence of comparable protein peaks in the vector-only control strains further validates the quantification of the secreted enzymes (see Additional file 1).

## Abbreviations

pHBAH, p-hydroxybenzoic acid hydrazide; DNS, 3,5-dinitrosalicylic acid; *Tl*XynA, *Thermomyces lanuginosus* xylanase A; *Tr*bMan, *Trichoderma reesei* beta-mannanase; *Tr*CBH2, *Trichoderma reesei* cellobiohydrolase 2; qRT-PCR, Quantitative real time polymerase chain reaction; P(GAP), Glyceraldehyde-3-phosphate dehydrogenase promoter; P(AOX1), Alcohol oxidase 1 promoter; PASC, Phosphoric acid-swollen cellulose; CMC, Carboxymethylcellulose.

## Competing interests

The authors declare that they have no competing interests.

## Authors’ contributions

AG conceived the project and KF managed the project and helped with the experimental implementation. AG and KF both helped to draft and improve the manuscript. AM carried out all experiments except of the bioreactor cultivations and drafted the manuscript. Bioreactor cultivations were performed and analyzed by RW. All authors have read and approved the final manuscript.

## Supplementary Material

Additional file 1 **Supplementary figures.** These figures provide virtual protein gels for each of the expressed enzymes at different time points during the fermentation runs and give a relative estimation of the purity of the expressed enzymes. Click here for file

Additional file 2 **These tables provide the individual data points of the measured target protein concentrations for the Figures**2,3**and**4. Click here for file

Additional file 3 **This figure provides a comparison of a triplicate measurement of a*Tr*CBH2 sample to exemplify the accurateness of the detection method.** Click here for file

Additional file 4 **Deglycosylation of *Tr*bMan.** These figures provide a comparison of glycysylated and EndoH-deglycosylated protein samples of *Tr*bMan. Click here for file
