# Supplementary material for: Expression of lignocellulolytic enzymes in Pichia pastoris
Source: Microb Cell Fact. 2012 May 14;11:61. doi: 10.1186/1475-2859-11-61 (PMC3503753; doi:10.1186/1475-2859-11-61)
Supplement: Additional file 1 — Supplementary figures. These figures provide virtual protein gels for each of the expressed enzymes at different time points during the fermentation runs and give a relative estimation of the purity of the expressed enzymes. [file 1475-2859-11-61-S1.pdf]

Supplementary figures

These figures provide quantitative and time-related protein expression data which were determined by micro-fluidic capillary electrophoresis using a Labchip GXII microcapillary system (Caliper Life Sciences, Hopkinton, USA; now Perkin Elmer). The virtual protein gels for each of the expressed enzymes at different time points during the fermentation runs are derived from the individual electropherograms and give a relative estimation of the purity of the expressed enzymes. Proteins were quantified with this method by integration of the protein-specific peak areas in the electropherograms which were then normalized to an external reference protein (BSA) of known concentration. General note: A notable peak shift to higher molecular weights as compared to SDS-PAGE gels is observed using this detection method. According to Perkin Elmer this effect is a well known phenomenon for Labchip GXII measurements. The effect is matrix-dependent and is particularly observed with glycosylated proteins.

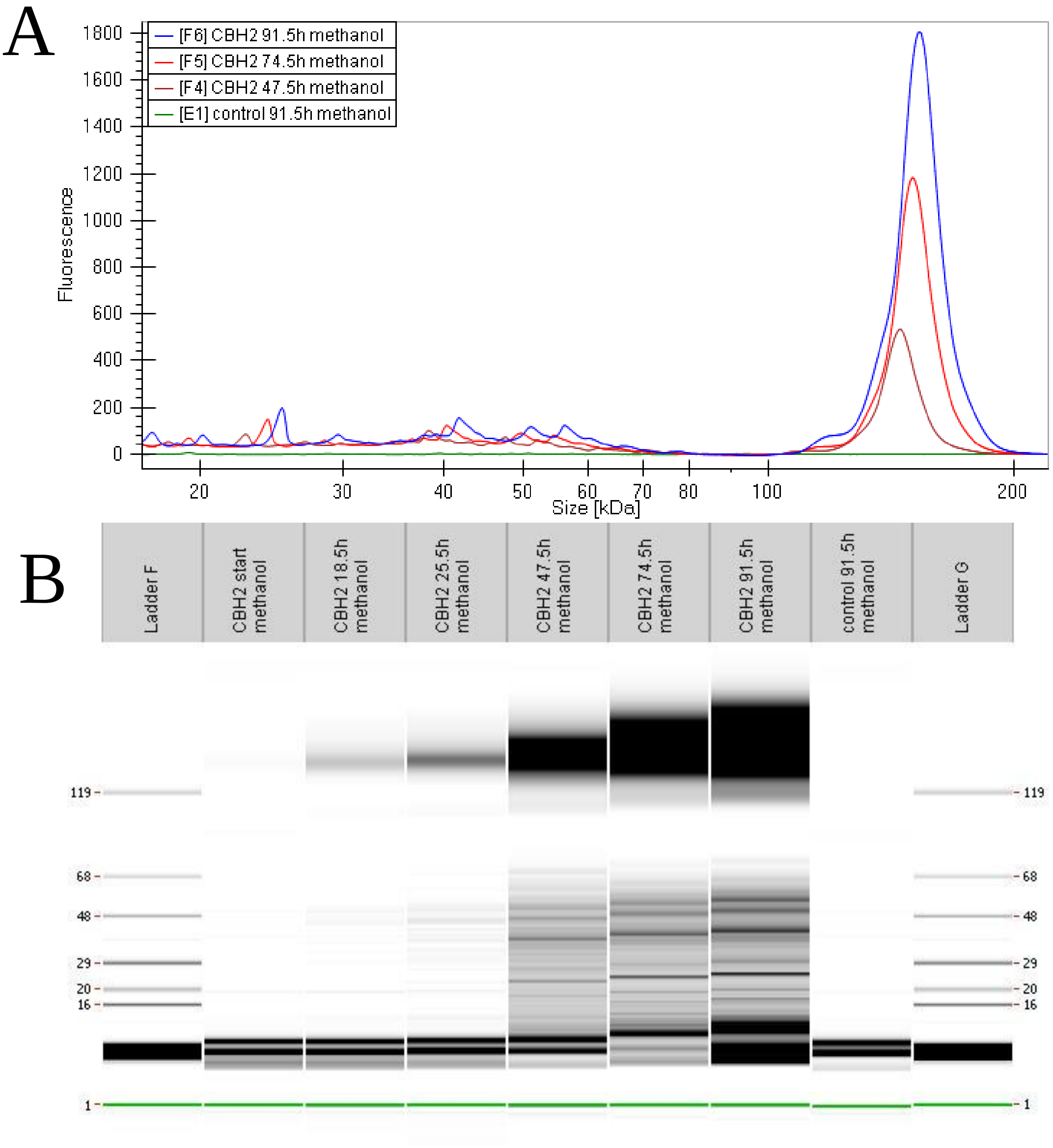

Figure 1: Electropherograms (A) and virtual gels (B) of heterologously expressed *Tr*CBH2. The supernatants of a *P. pastoris* strain producing *Tr*CBH2 at different time points

after induction were analyzed by capillary electrophoresis (Labchip GXII, Caliper Life Sciences, Hopkinton, USA) as described in the methods section. *Tr*CBH2 was produced under the control of the methanol inducible promoter P(En) and is detected at around 125 kDa. Panel A: Supernatants after 47.5, 74.5 and 91.5 h of induction with 0.5% methanol and a negative control (empty vector control) after 91.5 h. Panel B: Lane 1 and 9: Ladder; Lane 2 to 7: supernatants after 0, 18.5, 25.5, 47.5, 74.5, 91.5 h of induction start. Lane 8: supernatant of a negative control (empty vector control) after 91.5h of induction with 0.5% methanol.

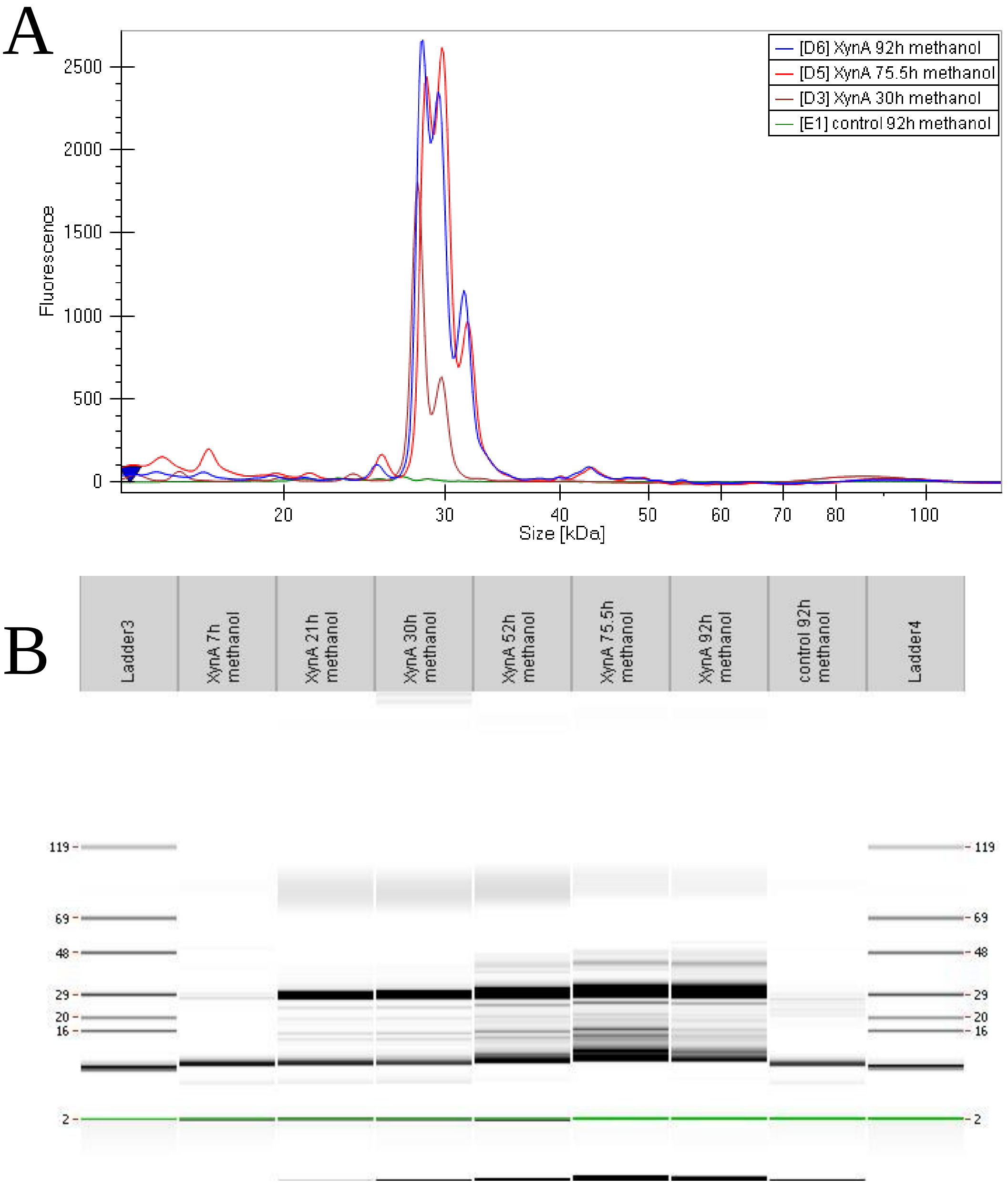

Figure 2: Electropherograms (A) and virtual gels (B) of heterologously expressed *Tl*XynA. The supernatants of a *P. pastoris* strain producing *Tl*XynA at different time points after induction were analyzed by capillary electrophoresis (Labchip GXII, Caliper Life Sciences, Hopkinton, USA) as described in the methods section. *Tl*XynA was produced under the control of the methanol inducible promoter P(En) and is detected at around 30 kDa. Panel A: Supernatants after 30, 75.5 and 92 h of induction with 0.5% methanol and a negative control (empty vector control) after 92 h. Panel B: Lane 1 and 9: Ladder; Lane 2 to 7: supernatants after 7, 21, 30, 52, 75.5, 92 h of induction start. Lane 8: supernatant of a negative

control (empty vector control) after 92h of induction with 0.5% methanol. Note: The inhomogeneous peak pattern of the electropherogram is due to inhomogeneous glycosylation.

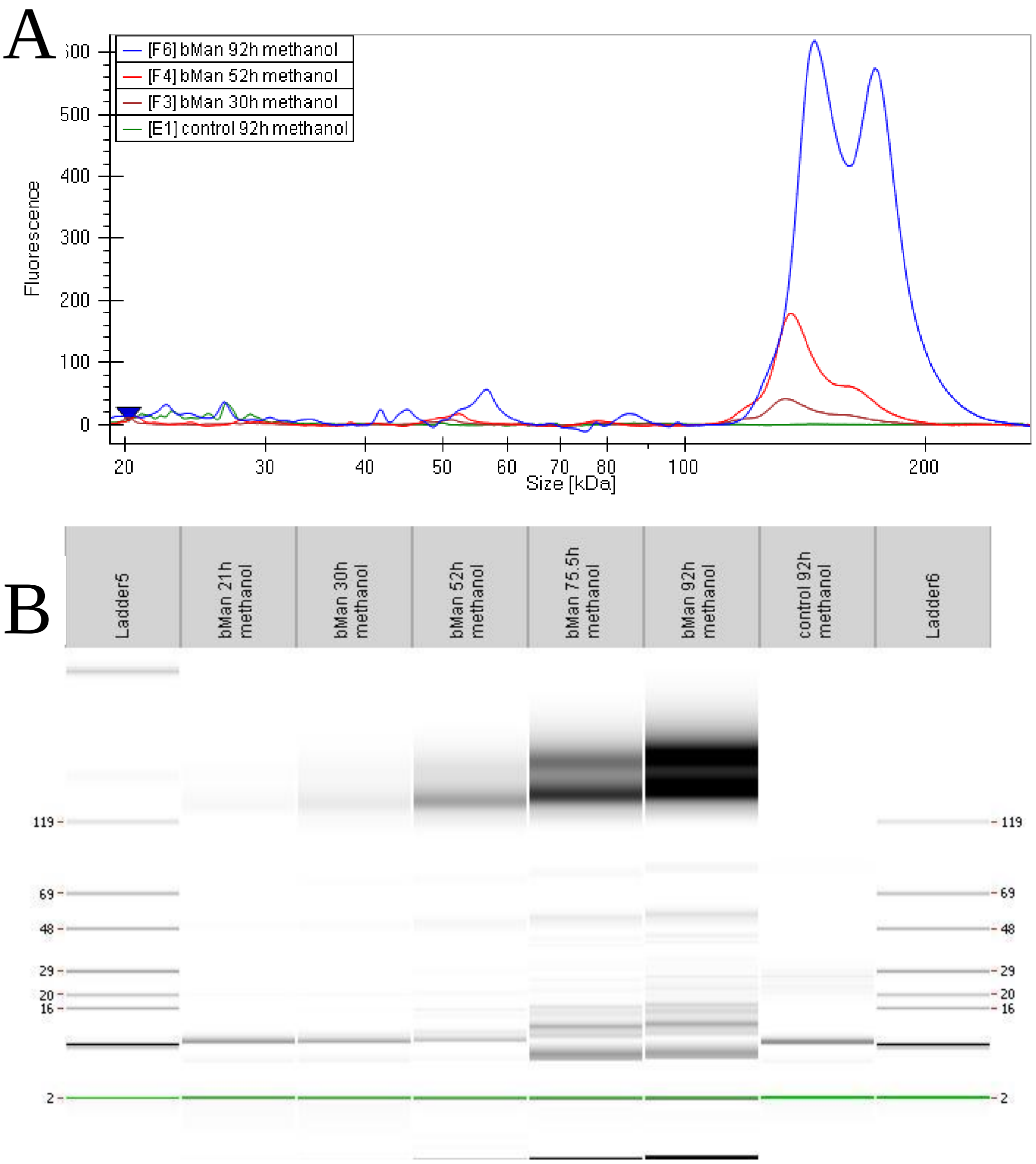

Figure 3: Electropherograms (A) and virtual gels (B) of heterologously expressed *Tr*bMan. The supernatants of a *P. pastoris* strain producing *Tr*bMan at different time points after induction were analyzed by capillary electrophoresis (Labchip GXII, Caliper Life Sciences, Hopkinton, USA) as described in the methods section. *Tr*bMan was produced under the control of the derepressed promoter P(De) and is detected at around 130 kDa. Panel A: Supernatants after 30, 52 and 92 h of induction with 0.5% methanol and a negative control (empty vector control) after 92 h. Panel B: Lane 1 and 8: Ladder; Lane 2 to 6: supernatants after 21, 30, 52, 75.5 and 92 h of induction start. Lane 8: supernatant of a negative control (empty vector control) after 92h of induction with 0.5% methanol. Note: The observed double peak pattern of the electropherogram is due to inhomogeneous glycosylation pattern.
