# Supplementary material for: Expression of lignocellulolytic enzymes in Pichia pastoris
Source: Microb Cell Fact. 2012 May 14;11:61. doi: 10.1186/1475-2859-11-61 (PMC3503753; doi:10.1186/1475-2859-11-61)
Supplement: Additional file 2 — These tables provide the individual data points of the measured target protein concentrations for the Figures2,3and4. [file 1475-2859-11-61-S2.pdf]

Deglycosylation of *Tr*bMan

This figure provides the analysis of *Endo*H treated or non treated supernatants of a *P. pastoris* strain expressing *Tr*bMan. The data was determined by micro-fluidic capillary electrophoresis using a Labchip GXII microcapillary system (Caliper Life Sciences, Hopkinton, USA).

A

B

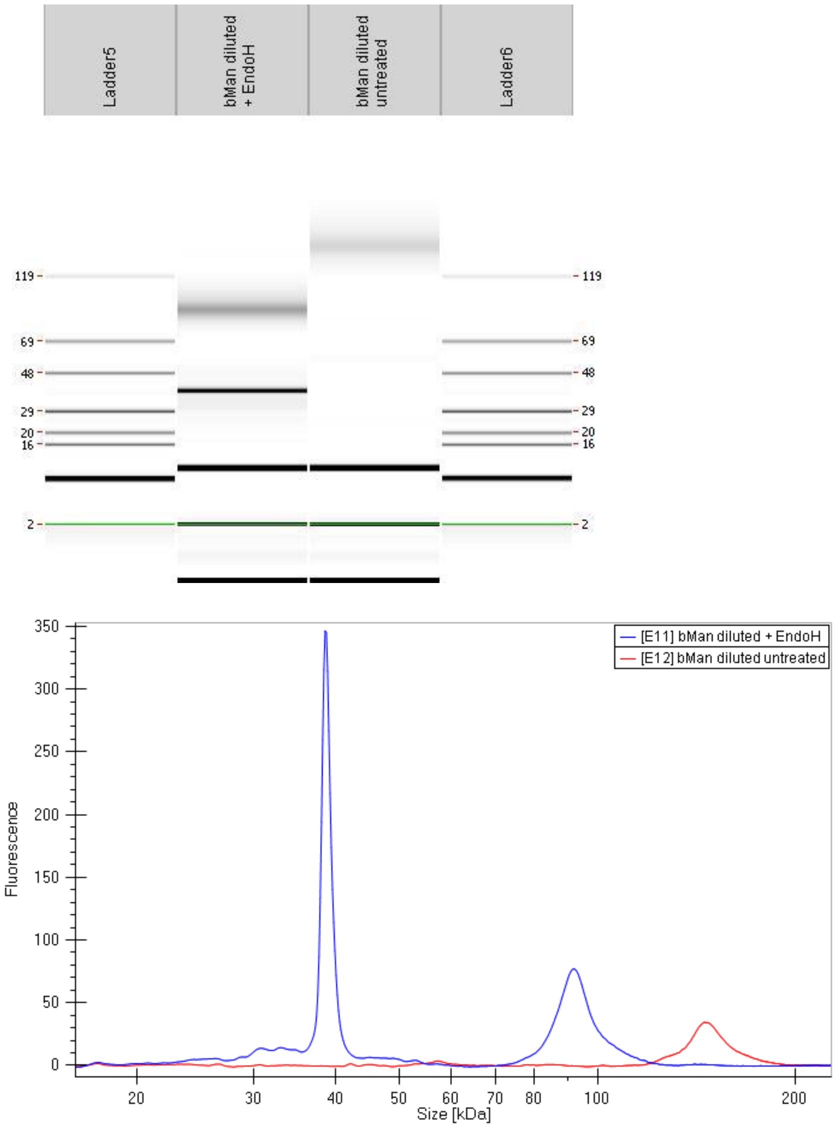

Figure 1: Electropherograms (A) and virtual gels (B) of *Endo*H treated or non treated *Tr*bMan.

*Endo*H treated or non treated supernatants of a *P. pastoris* strain producing *Tr*bMan were analyzed by capillary electrophoresis (Labchip GXII, Caliper Life Sciences, Hopkinton, USA) as described in the methods section. The reduction of MW due to deglycosylation is clearly visible in the virtual protein gel as well as in the electropherogram. The glycosylated *Tr*bMan (red line) was detected at around 130 kDa whereas the deglycosylated protein is detected around 90kDa (blue line). The peak area around 40kDa (blue line) represents *Endo*H.
