# Supplementary material for: Expression of lignocellulolytic enzymes in Pichia pastoris
Source: Microb Cell Fact. 2012 May 14;11:61. doi: 10.1186/1475-2859-11-61 (PMC3503753; doi:10.1186/1475-2859-11-61)
Supplement: Additional file 3 — This figure provides a comparison of a triplicate measurement of aTrCBH2 sample to exemplify the accurateness of the detection method. [file 1475-2859-11-61-S3.pdf]

Supplementary figures/ additional file 3

This figure provides a comparison of a triplicate measurement of a *Tr*CBH2 sample to show the usually relative low standard deviation of below 10 % of the detection system. Protein concentrations were determined by micro-fluidic capillary electrophoresis using a Labchip GXII microcapillary system (Caliper Life Sciences, Hopkinton, USA).

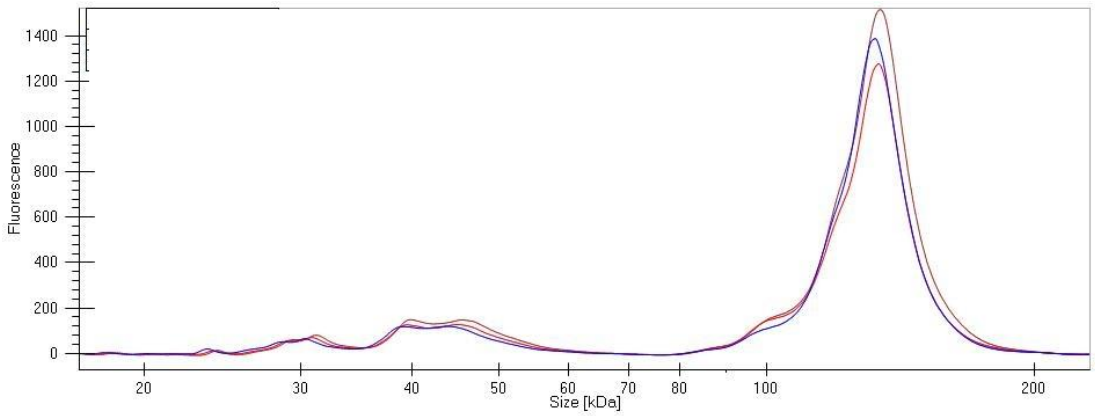

Figure 1: Electropherograms of heterologously expressed *Tr*CBH2 measured as a triplicate.

The supernatant of a *P. pastoris* strain producing *Tr*CBH2 under the control of P(De) at 90 h of induction was analyzed in triplicates to show the relative low standard deviation by capillary electrophoresis (Labchip GXII, Caliper Life Sciences, Hopkinton, USA) as described in the methods section. The obtained values are listed in Table 1.

Table 1: Triplicate measurement of protein concentration, calibrated against BSA as external standard. Data represent the measured protein concentrations of *Tr*CBH2 under the control of P(De) at 90 h of induction, measured in triplicate.

	protein concentration (g/l)	mean protein concentration (g/l)	coefficient of variation (%)
Peak 1	4,51	4,38	2.88
Peak 2	4,36		
Peak 3	4,26		
