# Supplementary material for: Expression of lignocellulolytic enzymes in Pichia pastoris
Source: Microb Cell Fact. 2012 May 14;11:61. doi: 10.1186/1475-2859-11-61 (PMC3503753; doi:10.1186/1475-2859-11-61)
Supplement: Additional file 4 — Deglycosylation of TrbMan. These figures provide a comparison of glycysylated and EndoH-deglycosylated protein samples of TrbMan. [file 1475-2859-11-61-S4.pdf]

Additional file 4

These tables provide the individual data points of the measured target protein concentration for the Figures 2, 3 and 4. For the explanation of the different abbreviations please refer to the original Figure legends.

Table 1: Measured protein concentrations during fed-batch cultivations of *Tr*CBH2 variants, optimized by different gene optimization methods; data as represented in Figure 2A.

Time (h)	P(AOX1)-*Tr*CBH2-CP-CN7±1 (g/l)	P(De)-*Tr*CBH2-CP-CN25±7 (g/l)	P(AOX1)-*Tr*CBH2-HM-CN3 (g/l)	P(De)-*Tr*CBH2-HM-CN7±1 (g/l)	P(De)-*Tr*CBH2-HM-CN1 (g/l)	P(GAP)-*Tr*CBH2-HM-CN4 (g/l)
0	0	0,01	0	0	0	0
18	0	0,22	0,05	0,14	0,01	0,35
47	0,18	0,73	0,81	1,20	0,05	1,11
74	0,69	2,42	1,70	2,74	0,35	1,70
90	1,26	3,81	2,78	4,37	0,43	2,43

Table 2: Measured protein concentrations during fed-batch cultivations of *Tr*CBH2 variants, optimized by different gene optimization methods; and normalized to regarding copy numbers, as represented in Figure 2B.

Time (h)	P(AOX1)-*Tr*CBH2-CP-CN7±1 (g/l)	P(De)-*Tr*CBH2-CP-CN25±7 (g/l)	P(AOX1)-*Tr*CBH2-HM-CN3 (g/l)	P(De)-*Tr*CBH2-HM-CN7±1 (g/l)	P(De)-*Tr*CBH2-HM-CN1 (g/l)	P(GAP)-*Tr*CBH2-HM-CN4 (g/l)
0	0	0	0	0	0	0
18	0	0,01	0,02	0,02	0,01	0,09
47	0,03	0,03	0,27	0,17	0,05	0,28
74	0,10	0,10	0,57	0,39	0,35	0,42
90	0,18	0,15	0,93	0,63	0,43	0,61

Table 3: Measured protein concentrations during fed-batch cultivations of *Tr*bMan and *Tl*XynA; data as represented in Figure 2A.

Time (h)	P(En)-*Tl*XynA-HM-CN10±3 (g/l)	P(En)-*Tl*XynA-HM-CN18±4 (g/l)
0	0	0
13	0,20	0
20	0,35	0,10
40	0,40	0,20
66,5	0,60	0,20

Time (h)	P(En)-*Tl*XynA-HM-CN6±1 (g/l)
0	0
21	0,26
30	0,37
52	0,61
75,5	0,86
92	0,85

Time (h)	P(En)-*Tr*bMan-HM-CN1 (g/l)
0	0
20	0,05
26	0,08
52	0,25
72	0,68
91	0,71

Table 4: Individual data points of the fed- batch cultivation of selected strains; data points at 90 h and the respective copy numbers and expression levels normalized to copy number; data as represented in Figure 3.

Strain	*Tr*CBH2$_{t90}$ (g/l)	CN	*Tr*CBH2$_{t90}$/CN (g/l)
P(AOX1)-CP-CN7±1	1,26	7	0,18
P(De)-CP-CN25±7	3,81	25	0,15
P(AOX1)-HM-CN3	2,78	3	0,93
P(De)-HM-CN7±1	4,37	7	0,63
P(De)-HM-CN1	0,43	1	0,43
P(GAP)-HM-CN4	2,43	4	0,61

Table 5: Relative ratios of the normalized expression levels at 90 h of the different gene optimization variants (HM/CP) under the control of P(AOX1) and P(De), data as represented in Figure 3B:

Promoter	HM$_{t90, norm}$ (g/l)		CP$_{t90, norm}$ (g/l)	HM$_{t90, norm}$/CP$_{t90, norm}$
P(De)	0,63	0,43	0,15	3,46 ±0,92
P(AOX1)	0,93		0,18	5,14

Table 6: Relative ratios of the normalized expression levels at 90 h of the different methanol inducible promoters (P(AOX1)/P(De)) expressing either *Tr*CBH2-CP or *Tr*CBH2-HM, data as represented in Figure 3C:

Gene optimization method	P(AOX1) (g/l)	P(De) (g/l)		P(AOX1)/P(De)
CP$_{t90, norm}$	0,18	0,15		1,18
HM$_{t90, norm}$	0,93	0,63	0,43	1,76

Table 7: Measured protein concentrations during fed-batch cultivations of *Tr*CBH2 variants expressed under the control of strong methanol inducible promoters (P(AOX1), P(En)) compared to the weak methanol inducible promoter P(De), data as represented in Figure 4A

Time (h)	P(AOX1)-*Tr*CBH2-HM-CN5±1 (g/l)
0	0
20,5	0,34
28,5	0,90
41,5	2,63
55,5	4,19
70	5,00
91,5	5,98

Time (h)	P(En)-*Tr*CBH2-HM-CN6±1 (g/l)
0	0,05
19	0,31
27,5	2,08
44	5,11
51,5	4,90
67,5	6,55
90,5	5,55

Time (h)	P(De)-*Tr*CBH2-HM-CN7±1 (g/l)
0	0
18	0,14
47	1,20
74	2,74
90	4,37

Table 8: Measured protein concentrations during fed-batch cultivation of a *Tr*bMan variant compared to previously obtained results, data as represented in Figure 4B:

Time (h)	P(De)-TrbMan-HM-CN5±1 (g/l)	P(En)-TrbMan-HM-CN1 (g/l)
0	0	0
20	0,10	0,05
26	0,40	0,08
52	0,60	0,25
72	0,90	0,68
91	1,14	0,72
